# Effects of Long-Term Water-Aging on Novel Anti-Biofilm and Protein-Repellent Dental Composite

**DOI:** 10.3390/ijms18010186

**Published:** 2017-01-18

**Authors:** Ning Zhang, Ke Zhang, Mary A. S. Melo, Michael D. Weir, David J. Xu, Yuxing Bai, Hockin H. K. Xu

**Affiliations:** 1 Department of Orthodontics, School of Stomatology, Capital Medical University, Beijing 100050, China; dentistzhang112@163.com (N.Z.); tuzizhangke@163.com (K.Z.); 2 Department of Endodontics, Periodontics and Prosthodontics, University of Maryland Dental School, Baltimore, MD 21201, USA; mmelo@umaryland.edu (M.A.S.M.); mweir@umaryland.edu (M.D.W.); djxu334@gmail.com (D.J.X.); 3 Center for Stem Cell Biology & Regenerative Medicine, University of Maryland School of Medicine, Baltimore, MD 21201, USA; 4 Marlene and Stewart Greenebaum Cancer Center, University of Maryland School of Medicine, Baltimore, MD 21201, USA; 5 Department of Mechanical Engineering, University of Maryland, Baltimore County, MD 21250, USA

**Keywords:** dental composite, protein repellent, antibacterial, long-term durability, water-aging, caries inhibition

## Abstract

The aims of this study were to: (1) synthesize an anti-biofilm and protein-repellent dental composite by combining 2-methacryloyloxyethyl phosphorylcholine (MPC) with quaternary ammonium dimethylaminohexadecyl methacrylate (DMAHDM); and (2) evaluate the effects of water-aging for 180 days on protein resistance, bacteria-killing ability, and mechanical properties of MPC-DMAHDM composite. MPC and DMAHDM were added into a resin composite. Specimens were stored in distilled water at 37 °C for 1, 30, 90, and 180 days. Mechanical properties were measured in three-point flexure. Protein attachment onto the composite was evaluated by a micro bicinchoninic acid approach. An oral plaque microcosm biofilm model was employed to evaluate oral biofilm viability vs. water-aging time. Mechanical properties of the MPC-DMAHDM composite after 180-day immersion matched those of the commercial control composite. The composite with 3% MPC + 1.5% DMAHDM had much stronger resistance to protein adhesion than control (*p* < 0.05). MPC + DMAHDM achieved much stronger biofilm-eradicating effects than MPC or DMAHDM alone (*p* < 0.05). Biofilm colony-forming units on the 3% MPC + 1.5% DMAHDM composite were three orders of magnitude lower than commercial control. The protein-repellent and antibacterial effects were durable and showed no loss in water-aging from 1 to 180 days. The novel MPC-DMAHDM composite possessed strong and durable resistance to protein adhesion and potent bacteria-eradicating function, while matching the load-bearing ability of a commercial dental composite. The novel MPC-DMAHDM composite represents a promising means of suppressing oral plaque growth, acid production, and secondary caries.

## 1. Introduction

Polymeric dental composites are increasingly popular in tooth cavity restorations [1,2]. Resin compositions, curing and polymerization efficacy, and fracture resistance have been significantly improved [3,4,5,6,7]. However, studies have indicated that composites had more biofilm (plaque) accumulation than other restoratives [8,9]. Biofilms with acid production lead to recurrent caries, which is a primary reason for restoration failures [10,11]. Nearly 50% of dental restorations fail within 10 years [12], and repairing them amounts to up to 50% to 70% of a dentist’s time [12,13]. Hence, it is beneficial to synthesize a new kind of anti-biofilm composites that can prevent secondary caries.

An important approach involves the synthesis of quaternary ammonium monomers (QAMs) [14,15,16]. For example, 12-methacryloyloxydodecylpyridinium bromide (MDPB) [17,18,19,20,21,22] and methacryloxylethylcetyl dimethyl ammonium chloride (DMAE-CB) [23] could copolymerize in resins to impart an antibacterial function. Recently, a quaternary ammonium dimethacrylate (QADM) was added into a resin composite, which exhibited great anti-biofilm properties [24,25,26,27]. Previous experiments showed that the bacteria-eradicating property of QAMs increased with the alkyl chain length (CL) increasing from 5 to 16, and then declined when CL was further increased to 18 [28,29]. A new dimethylaminohexadecyl methacrylate (DMAHDM) with CL of 16 was recently shown to have the strongest antibacterial activity among the QAMs tested [29].

The second approach is to render the dental resin protein-repellent. After the composite is exposed in the oral cavity, proteins are adsorbed onto its surface, which serve as a prerequisite for bacteria attachment [30,31]. The adsorption of oral bacteria to the resin composite is mediated by the adsorbed proteins [30,31]. Biofilm is the source of infection with organic acids leading to caries [32]. Accordingly, it is meritorious for the dental composite to be able to resist proteins and deter bacterial attachment, thereby reducing biofilm formation and caries. It was shown that proteins tended to adhere to hydrophobic surfaces [33,34]. However, highly hydrophilic surface coatings using 2-methacryloyloxyethyl phosphorylcholine (MPC) can resist protein adsorption and bacterial adhesion [35,36,37]. MPC is a methacrylate with a phospholipid polar group in the side chain [38,39]. Various biomedical commercial products using MPC polymer have been synthesized and used clinically [35,36,37]. Recently, MPC was added into dental resins, achieving a great protein-repellent property [40]. Furthermore, MPC was combined with DMAHDM to synthesize a resin with a combination of antibacterial and protein-repellent abilities [41]. The novel MPC-DMAHDM containing composite greatly inhibited protein adsorption and bacterial viability without compromising the mechanical properties [41]. However, the long-term antibacterial and protein-repellent effects of the composite after water-aging had not been reported.

Therefore, the present study added MPC and DMAHDM in a dental composite and performed water-aging test for 180 days (d). An oral plaque microcosm model was used to incubate biofilms on the polymeric composite. The objectives of this study were to develop a novel composite with long-lasting anti-biofilm and protein-repellent capabilities. It was hypothesized that: (1) the anti-biofilm and protein-repellent capabilities of the MPC-DMAHDM composite would not decrease with increasing water-aging time; (2) the MPC-DMAHDM composite would greatly decrease protein adsorption and bacterial viability compared to a commercial composite, even after long-term water-aging; and (3) the MPC-DMAHDM composite would possess mechanical properties as good as a commercial control composite without antibacterial activity, even after long-term water-aging.

## 2. Results

**Figure 1:**
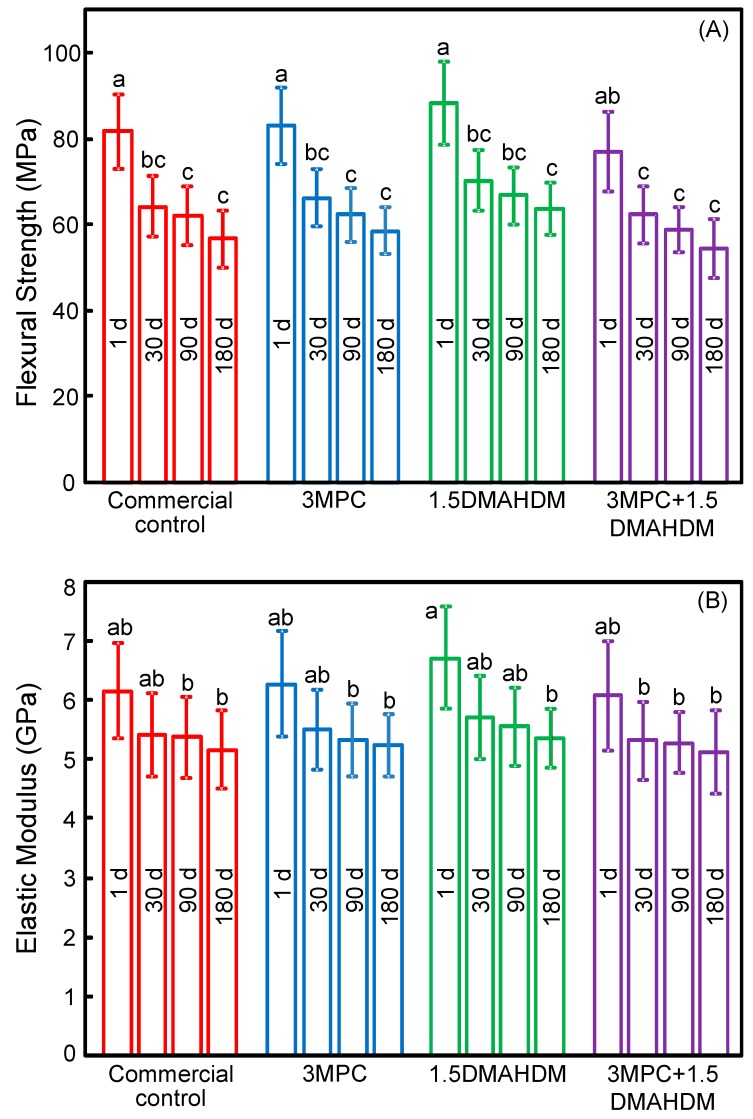
Mechanical properties of composites: (**A**) Flexural strength; and (**B**) elastic modulus (mean ± SD; *n* = 6). Strength and modulus of MPC-DMAHDM composite after 180-day (d) water-aging matched those of control composite. MPC, 2-methacryloyloxyethyl phosphorylcholine; DMAHDM, dimethylaminohexadecyl methacrylate. Bars with dissimilar letters indicate values that are significantly different from each other (*p* < 0.05).

Figure 1 plots (A) flexural strength; and (B) elastic modulus of the composites (mean ± SD; *n* = 6). Mechanical properties indicated a decrease during the first month of water-aging, with little further decrease observed from 30 to 180 days. After the 180 day immersion, the strength and modulus of the 3MPC + 1.5DMAHDM composite were similar to those of the commercial control (*p* > 0.1).

**Figure 2:**
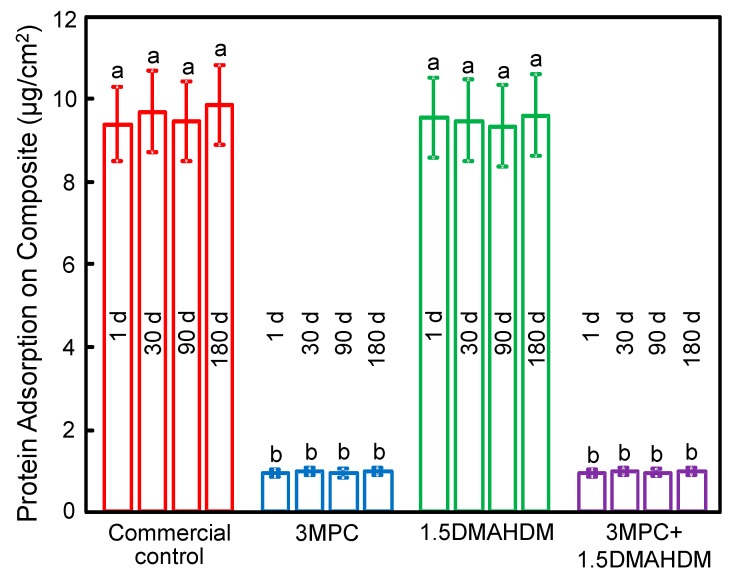
Protein adsorption on sample surfaces (mean ± SD; *n* = 6). The composite with 3% MPC + 1.5% DMAHDM had protein adsorption that was one tenth that of control (*p* < 0.05). There was no significant difference between 1 day (d) and 180 days, showing that the protein-repellency was not lost in water-aging. Bars with dissimilar letters indicate values that are significantly different from each other (*p* < 0.05).

Protein adsorption on samples is plotted in Figure 2 (mean ± SD; *n* = 6). Adding MPC into a composite greatly inhibited protein adsorption, and there was no difference between 1 day and 180 days. The 3MPC + 1.5DMAHDM composite had the same protein adsorption as that of the 3MPC composite (*p* > 0.1), which was about one tenth that of the control composite and the 1.5DMAHDM composite (*p*< 0.05).

**Figure 3:**
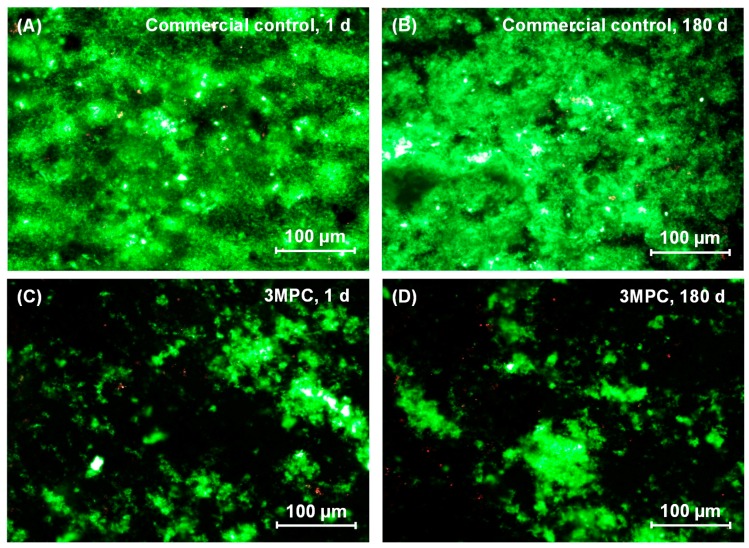

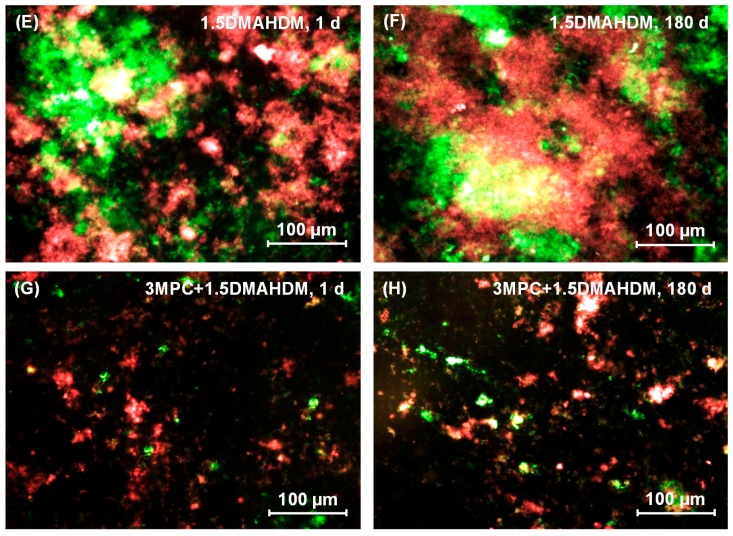
Representative live/dead images of two-day biofilms on composites: (**A**,**B**) Commercial control at 1 and 180 days (d), (**C**,**D**) 3MPC at 1 and 180 d, (**E**,**F**) 1.5DMAHDM at 1 and 180 d, and (**G**,**H**) 3MPC + 1.5DMAHDM at 1 and 180 d. Live bacteria were stained green, and dead bacteria were stained red. When live/dead bacteria were in close proximity of each other, the staining exhibited yellow/orange colors. In (**A**), the commercial control had mostly live bacteria. In contrast, in (**C**), the 3MPC composite had much less bacterial attachment. In (**E**), the composite with 1.5% DMAHDM had substantial amounts of dead bacteria with red staining. In (**G**), the 3MPC + 1.5DMAHDM composite had less bacterial coverage, and the biofilms consisted of mostly compromised bacteria. As shown in (**B**,**D**,**F**,**H**), there was no significant difference between 1 and 180 d.

Typical live/dead assay images of oral biofilms adhering on samples are shown in Figure 3. The commercial control was fully covered by live bacteria. In contrast, the 3MPC composite had little bacterial attachment. The composite with 1.5% DMAHDM had great antibacterial property, yielding lots of dead bacteria. The 3MPC + 1.5DMAHDM composite had less bacterial attachment, and the bacteria were mostly dead. There was no significant difference between 1 day and 180 days samples, showing that the protein-repellent and anti-biofilm function was not lost during 180 days of water-aging.

**Figure 4:**
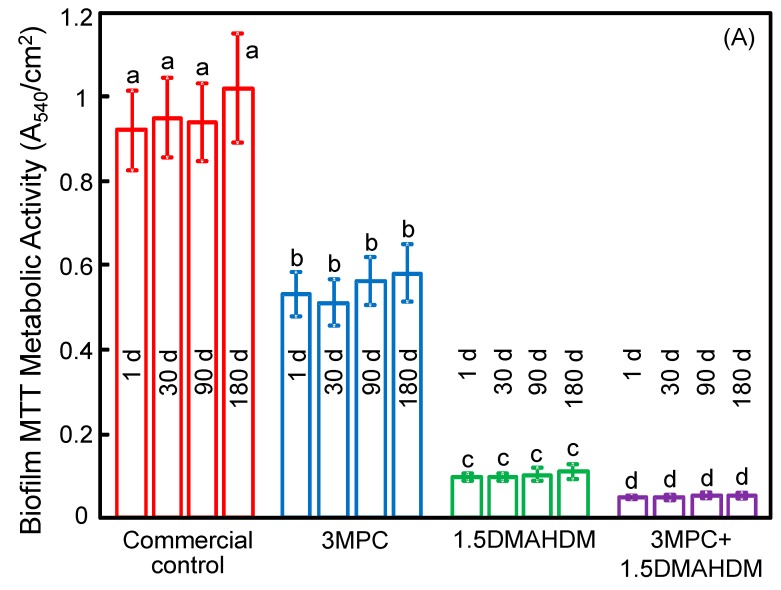

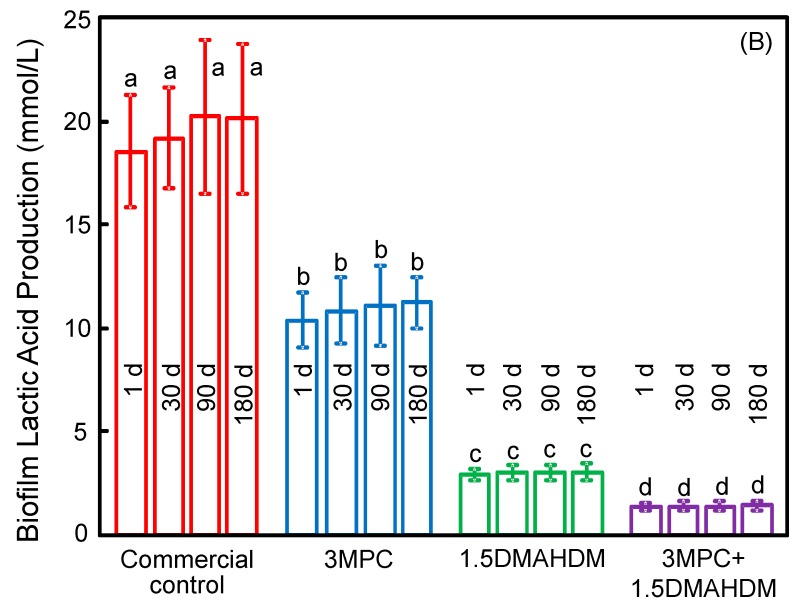
(**A**) Metabolic activity; and (**B**) lactic acid production (mean ± SD; *n* = 6). The composite with 3% MPC + 1.5% DMAHDM had the least metabolic activity and lactic acid production. Water-aging for 180 days (d) did not decrease the antibacterial function, compared to 1 day (*p* > 0.1). Bars with dissimilar letters indicate values that are significantly different from each other (*p* < 0.05).

Figure 4 plots: (A) metabolic activity, and (B) lactic acid production (mean ± SD; *n* = 6). Adding MPC or DMAHDM each alone strongly lowered the viability of biofilms, compared to the control (*p* < 0.05). The 3MPC + 1.5DMAHDM composite had the least metabolic activity and lactic acid production. Water-aging for six months did not decrease the antibacterial function, compared to one day (*p* > 0.1).

**Figure 5:**
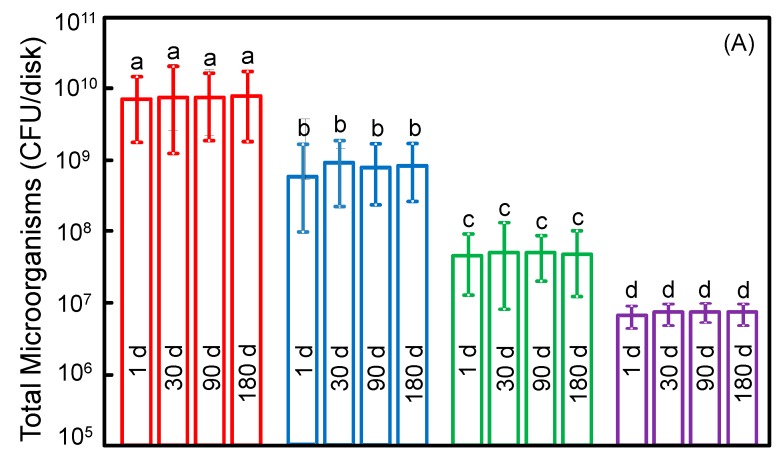

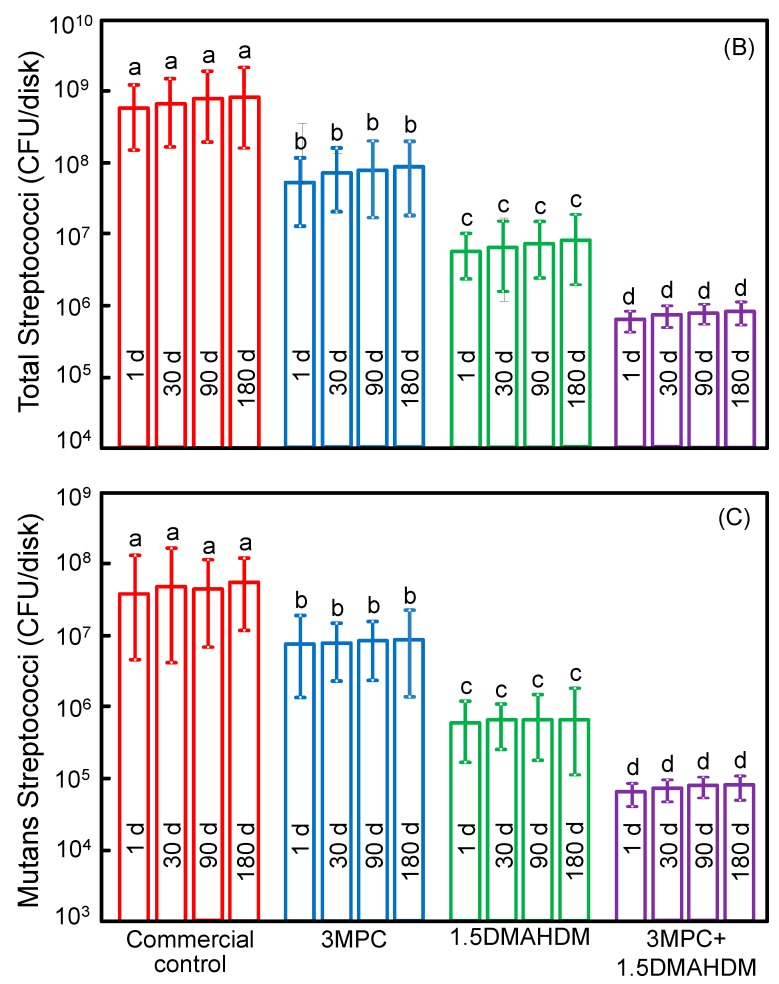
Colony-forming unit (CFU) counts for: (**A**) total microorganisms; (**B**) total streptococci; and (**C**) mutans streptococci (mean ± SD; *n* = 6). CFU counts on the 3MPC + 1.5DMAHDM composite were three orders of magnitude lower than commercial composite (*p* < 0.05). There was no significant difference of CFU before and after 180 days (d) (*p* > 0.1), demonstrating that the anti-biofilm function was not lost in long-term water-aging. Bars with dissimilar letters indicate values that are significantly different from each other (*p* < 0.05).

Figure 5 plots: (A) total microorganisms; (B) total streptococci; and (C) mutans streptococci (mean ± SD; *n* = 6). Incorporating MPC or DMAHDM each alone decreased the biofilm colony forming units (CFU), compared to the commercial control (*p* < 0.05). The 3MPC + 1.5DMAHDM composite had greater antibacterial properties than using MPC or DMAHDM alone (*p* < 0.05). There was no significant difference of CFU before and after six months of water-aging for each group (*p* > 0.1). All three CFU counts on 3MPC + 1.5DMAHDM composite were nearly three orders of magnitude lower than that of commercial control, both at 1 day and 180 days of water-aging.

## 3. Discussion

This study evaluated the effects of water-aging for six months on the mechanical properties and the durability of antibacterial and protein-repellent activities of the novel MPC-DMAHDM composite. The hypotheses were proven in that the antibacterial and protein-repellent properties of MPC-DMAHDM composite did not decline with increasing the water-aging time; the MPC-DMAHDM composite greatly inhibited protein attachment and bacterial viability compared to control composite, even after long-term water-aging, while the mechanical properties matched those of the commercial composite control. The polymeric composite using 3% MPC and 1.5% DMAHDM decreased biofilm CFU by three orders of magnitude, compared to commercial composite control, both before and after water-aging for six months. These results demonstrate that the protein-repellent and antibacterial functions were maintained during long-term water-aging.

The MPC incorporation into the composite had three merits. First, it was shown that most proteins tended to adhere onto hydrophobic material surfaces [33,34]. The MPC polymer was hydrophilic, prevented the adsorption of proteins, and inhibited the attachment of bacteria [35,36,37]. Regarding the mechanism of protein-repellency, it was reported that MPC is hydrophilic [38] and that there is lots of free water but no bound water in the hydrated MPC polymer [39]. The bound water would lead to protein attachment [39,42]. In contrast, the free water around the phosphorylcholine group could repel proteins effectively, thus inhibiting proteins [39,43]. The present study showed that mixing MPC into a polymeric composite resulted in a composite that greatly resisted protein adhesion (Figure 2). Because MPC diminished protein adsorption, it resisted bacterial adhesion and biofilm formation. Second, one drawback of resin composites containing QAM is that the attachment of salivary proteins on composite surfaces could reduce the efficacy of “contact-inhibition”, thus decreasing the anti-biofilm potency [44,45]. Because MPC can strongly resist protein attachment, leading to much less proteins attaching on the composite and therefore more direct resin-bacteria contact, the contact-killing effect is thus facilitated and enhanced. Indeed, the results in Figure 3, Figure 4 and Figure 5 confirmed that the 3MPC + 1.5DMAHDM composite had greater antibacterial properties than using MPC or DMAHDM alone. Third, MPC has reactive methacrylate groups that can be co-polymerized and covalently bonded with the resin matrix via photo polymerization [46]. A previous experiment investigated the durability and capability to resist proteins for acrylic resin containing MPC. The results indicated that MPC was co-polymerized with acrylic resin through covalent bonding, providing long-lasting and durable prevention to protein attachment [46]. In addition, it was reported that the MPC-containing surface layer was resistant to mechanical stresses [47]. Furthermore, another study found that the MPC-modified layer offered high lubricity for the surface [48]. This lubrication may lead to persistence against the mechanical stress originated by brushing, thus providing enough durability for clinical applications [48]. In this study, MPC was copolymerized with the resin, resulting in MPC immobilization in the composite. The MPC was added into and co-polymerized with the entire volume of the resin composite, therefore, MPC was present even after brushing and wear to continue to deter protein adhesion. The results confirmed that adding MPC into a composite reduced protein attachment, and there was no significant difference between 1 day and 180 days, showing that the protein-repellent ability was not lost in water-aging.

The anti-biofilm mechanism of QAMs is that quaternary ammonium can lead to bacteria lysis by adhering to the cell membrane to produce cytoplasmic leakage [49,50]. When the bacterial cell touches the QAM resin, the electric balance of the cell membrane could be affected, resulting in cell death [49,50]. Long cationic polymers could infiltrate bacterial cells to affect membranes, like a needle bursting a balloon [51]. The present study indicated that DMAHDM indeed imparted a great anti-biofilm property to the composite. Regarding the antibacterial durability, previous studies indicated that adding QAMs in resins yielded a long-term anti-biofilm property, because the QAM was immobilized with the resin via covalent bonding with the polymer network [19,21]. Hence, the anti-biofilm QAM was copolymerized in the resin matrix, and was not released over time [17,18,21]. Previous studies have confirmed the long-term durability of QAM composites and adhesives [18,20,26,52]. For example, a study on a MDPB monomer, a bromide monomethacrylate, indicated that the anti-biofilm ability was maintained after the composite was stored in water for three months [52]. Another study on a QADM-containing composite indicated that the antibacterial properties had no significant decrease in water-aging from one day to six months [26]. These results are consistent with the present study indicating that the MPC-DMAHDM composite possessed durable protein-repellent and antibacterial effects. It should be noted that the live/dead images still showed some live bacteria (Figure 3G,H). Indeed, the metabolic activity and the lactic acid production of biofilms were reduced by the MPC-DMAHDM composite but did not reach 0 (Figure 4), and the total microorganisms CFU was reduced from 10^10^ to 10^7^, but was not lowered to approach 0. These results together showed that there were still some live bacteria on the MPC-DMAHDM composite. Therefore, further study is needed to enhance the potency of the bioactive composite and reduce the lactic acid production to be close to 0 to protect the teeth.

In addition to bacteria-killing ability and resistance to protein adhesion, it is imp.cortant for the composite to have load-bearing properties. Composites function in the oral enviro1nment which could lead to composite degradation [1,53,54]. Water-aging could affect the fillers [55], soften the resin because of the plasticizing action of water [56], and lead to hydrolytic breakdown of the interfaces between the fillers and the resin matrix [1,56]. Previous reports suggested that the composite should have both stable reinforcement fillers and ion-releasing fillers, to obtain both antimicrobial and load-bearing capabilities [57,58,59]. The key is that the composite should not rely on fillers that release ions to provide mechanical properties [59]. Instead, it should rely on strong and non-releasing fillers for mechanical properties [59]. In the present study, the MPC-DMAHDM composite contained 70% silanized glass fillers for reinforcement. As a result, the MPC-DMAHDM composite, while reducing biofilm CFU by three orders of magnitude, had mechanical properties similar to the control composite (Heliomolar) after 180-day immersion. Heliomolar is indicated for Classes I and II posterior restorations and Classes III and IV anterior restorations. Therefore, the novel MPC-DMAHDM composite with similar mechanical properties may also be suitable for these applications, with additional resistance to protein adhesion and bacteria-killing benefits. Further experiments are needed to investigate the long-term durability of this novel composite under in vivo conditions. In particular, repeated-challenge experiments are needed in which a biofilm is grown on the composite and then removed by actions such as tooth-brushing, and then new bacteria are inoculated on the composite to form new biofilms. Then this process is repeated many times to investigate the durability of the MPC-DMAHDM composite in reducing biofilm growth and acid production under repeated biofilm challenges.

## 4. Materials and Methods

### 4.1. Incorporation of Protein-Repellent MPC

MPC was obtained commercially (Sigma-Aldrich, St. Louis, MO, USA) which was synthesized via an approach reported by Ishihara et al. [38]. BisGMA (bisphenol glycidyl dimethacrylate) and TEGDMA (triethylene glycol dimethacrylate) (Esstech, Essington, PA, USA) were mixed at a mass ratio of 1:1, as a model resin. It was made light-curable by using 0.2% camphorquinone and 0.8% ethyl 4-*N*,*N*-dimethylaminobenzoate (mass fractions). It should be noted that the MPC and DMAHDM incorporation method can be applied to other dental resin systems. The MPC was added into the photo-activated BisGMA-TEGDMA resin (referred to as BT) at MPC/(BT + MPC) mass fraction of 10% [41]. Previous experiments showed that this mass fraction achieved a great protein-repellency without significantly compromising the mechanical strength of the final composite [40,41].

### 4.2. Incorporation of Antibacterial DMAHDM

DMAHDM was developed using a modified Menschutkin reaction [14,29], in which a tertiary amine group was reacted with an organo-halide. Briefly, 10 mmol of 2-(dimethylamino) ethyl methacrylate (DMAEMA, Sigma-Aldrich) and 10 mmol of 1-bromohexadecane (BHD, TCI America, Port-land, OR, USA) were combined with 3 g of ethanol in a 20 mL scintillation vial. The vial was stirred at 70 °C for 24 h. The solvent was then evaporated, obtaining DMAHDM as a clear, colorless, and viscous liquid [14,29]. DMAHDM was incorporated into the BT resin at a DMAHDM/(BT + DMAHDM) mass fraction of 5% [41]. Higher DMAHDM mass fractions were not employed because of a decrease in the mechanical properties when DMAHDM was combined with 10% MPC in the composite in our preliminary experiment.

### 4.3. Preparation of MPC-DMAHDM Composite

A barium boroaluminosilicate glass with a mean particle size of 1.4 µm (Caulk/Dentsply, Milford, DE, USA) was silanized with 4% 3-methacryloxypropyltrimethoxysilane and 2% *n*-propylamine [57]. The glass particles were incorporated into a resin at a filler mass fraction of 70% to form a cohesive paste. Because the resin mass fraction was 30%, the MPC mass fraction in the composite was 3%, and the DMAHDM mass fraction in the composite was 1.5%. As a commercial control composite, Heliomolar was used (Heliomolar, Ivoclar, ON, Canada). Heliomolar contained 66.7% silica and ytterbium-trifluoride fillers. The reason for selecting this commercial material was that it has fluoride release, and fluoride ions could inhibit bacterial growth. The purpose was for the new MPC-DMAHDM composite to have an antibacterial potency to be orders of magnitude stronger than that of a fluoride-releasing composite. Therefore, the following four composites were tested:

(1) Commercial composite control (Heliomolar);
(2) Protein-repellent composite: 70% glass + 27% BT + 3% MPC (termed “3MPC”);
(3) Antibacterial composite: 70% glass + 28.5% BT + 1.5% DMAHDM (termed “1.5DMAHDM”);
(4) Protein-repellent and antibacterial composite: 70% glass + 25.5% BT + 3% MPC + 1.5% DMAHDM (termed “3MPC + 1.5DMAHDM”).

These composites were water-aged for 1 to 180 days, hence the 1 day immersion also served as a control for each composite that was water-aged for various periods of time.

For mechanical measurements, each paste was placed into rectangular molds of 2 × 2 × 25 mm. For protein adhesion and oral biofilm experiments, each paste was put into disk molds of 9 mm in diameter and 2 mm in thickness. The samples were light-cured (Triad 2000, Dentsply, York, PA, USA) for 1 min on each side [26,27]. Six samples per composite were used for each test. Samples were stored in distilled water at 37 °C for 1, 30, 90, and 180 days. Each group was immersed in 200 mL of distilled water in a sealed polyethylene container, following previous experiments [58,59,60]. The water was changed once every week. The water-aged disks for protein adsorption and biofilm experiments were sterilized with ethylene oxide (Anprolene AN 74i, Andersen, Haw River, NC, USA) and de-gassed for three days before testing [29,50].

### 4.4. Mechanical Properties

At the end of each time period, the immersed samples were tested as soon as possible after being taken out of the water. A computer-controlled Universal Testing Machine (5500R, MTS, Cary, NC, USA) was used for three-point flexure with a 10 mm span at a crosshead speed of 1 mm/min [26,27]. Flexural strength (*S*) was calculated as: *S* = 3*P*_max_*L*/(2*bh*^2^), where P is the fracture load, L is span, b is specimen width, and h is thickness. Elastic modulus (*E*) was calculated as: *E* = (*P*/*d*)(*L*^3^/[4*bh*^3^]), where load *P* divided by displacement d is the slope in the linear elastic region [26,27].

### 4.5. Measurement of Protein Adsorption

Protein adsorbed on samples was evaluated by employing a micro bicinchoninic acid (BCA) method [36,37,40,41]. Each sample was placed in phosphate buffered saline (PBS) for 2 h before being immersed in 4.5 g/L bovine serum albumin (BSA) (Sigma-Aldrich) solutions at 37 °C for 2 h [36,37]. The samples then were washed with fresh PBS by stirring at a speed of 300 rpm for 5 min (Bellco Glass, Vineland, NJ, USA), stored in sodium dodecyl sulfate (SDS) at 1 wt % in PBS, and sonicated at room temperature for 20 min to completely remove the BSA from sample surfaces [40,41]. A protein analysis kit (micro BCA protein assay kit, Fisher Scientific, Pittsburgh, PA, USA) was employed to measure the BSA concentration in the SDS solution, from which the amount of protein adsorbed on the resin disk was then calculated [36,37,40,41].

### 4.6. Dental Plaque Microcosm Biofilm Model

The oral biofilm model was approved by the University of Maryland Institutional Review Board. Saliva is ideal for incubating oral microcosm biofilms in vitro, with the advantage of having much of the complexity and heterogeneity of the dental plaque in vivo [61]. Saliva was obtained from ten healthy human donors, who did not brush teeth for 24 h with no food intake for 2 h prior to donating saliva [26,27]. The saliva was diluted in sterile glycerol to a concentration of 70%, and stored at −80 °C for subsequent experiments [62].

The saliva-glycerol stock was added into the growth medium with 1:50 final dilution. The growth medium contained mucin (type II, porcine, gastric) at a concentration of 2.5 g/L; yeast extract, 1.0 g/L; tryptone, 2.0 g/L; bacteriological peptone, 2.0 g/L; KCl, 0.2 g/L; NaCl, 0.35 g/L; CaCl_2_, 0.2 g/L; cysteine hydrochloride, 0.1 g/L; vitamin K1, 0.0002 g/L; hemin, 0.001 g/L, at pH 7 [63]. Samples were sterilized in ethylene oxide (Anprolene AN 74i, Andersen, Haw River, NC, USA). Next, 1.5 mL of inoculum was added into each well of 24-well plates with a sample, and incubated for 8 h at 37 °C in 5% CO_2_. Then, the disks were transferred to new 24-well plates filled with fresh medium and incubated for 16 h, the disks were transferred to new 24-well plates with fresh medium and incubated for 24 h. This totaled 48 h of culture, which formed mature biofilms on resins, as shown previously [62].

### 4.7. Live/Dead Assay

Samples with two-day biofilms were rinsed with PBS and stained using the BacLight live/dead kit (Molecular Probes, Eugene, OR, USA) [26,27,40,41]. Live bacteria were stained green, and dead bacteria were stained red. The stained disks were determined using an inverted epifluorescence microscope (Eclipse TE2000-S, Nikon, Melville, NY, USA).

### 4.8. MTT (3-[4,5-Dimethylthiazol-2-yl]-2,5-Diphenyltetrazolium Bromide) Assay of Metabolic Activity

The MTT assay was used to determine the metabolic property of oral plaque biofilms [26,27,40]. Disks with oral biofilms that had grown for two days were placed in a new 24-well plate. One mL of MTT dye (0.5 mg/mL) was added into each well and incubated for 1 h at 37 °C in 5% CO_2_. Then, the samples were moved to a new 24-well plate, and 1 mL of dimethyl sulfoxide (DMSO) was added to solubilize the formazan crystals. After incubating for 20 min with moderate shaking in a darkroom, 200 µL of the DMSO solution from each well was collected, and its absorbance at 540 nm was evaluated via a microplate reader (SpectraMax M5, Molecular Devices, Sunnyvale, CA, USA).

### 4.9. Lactic Acid Production and Colony-Forming Unit (CFU) Counts

Samples with two-day biofilms were washed with cysteine peptone water (CPW) to remove loose bacteria [24,25]. They were placed onto 24-well plates with buffered peptone water (BPW) plus 0.2% sucrose. The samples were incubated for 3 h in 5% CO_2_ at 37 °C to let the biofilms to produce acid [24,25]. The BPW solutions were then stored for lactate analysis. Lactate concentrations in the BPW solutions were evaluated using an enzymatic (lactate dehydrogenase) method [24,25]. The microplate reader was used to measure the absorbance at 340 nm (optical density OD_340_) for the collected BPW solutions. Standard curves were obtained using a lactic acid standard (Supelco, Bellefonte, PA, USA) [24,25].

Samples with oral biofilms grown for two days were put into tubes with 2 mL CPW, and the biofilms were obtained by a sonication and vortexing process (Fisher, Pittsburgh, PA, USA) [26,27]. Three kinds of agar plates were employed to determine the CFU counts to evaluate the microorganism activity. First, tryptic soy blood agar culture plates were used to assess total microorganisms [63]. Second, mitis salivarius agar (MSA) culture plates with 15% sucrose added were employed to evaluate total streptococci [64]. Third, MSA agar culture plates containing 0.2 units of bacitracin per mL were used to assess mutans streptococci [63]. The bacterial suspensions were serially diluted and spread onto agar plates for CFU analysis, as described in previous reports [26,27].

### 4.10. Statistical Analysis

One-way and two-way analyses of variance (ANOVA) were employed to determine the significant effects of the experimental variables. Tukey’s multiple comparison test was used to compare the data at a *p*-value of 0.05.

## 5. Conclusions

A novel MPC-DMAHDM dental composite was synthesized with a strong resistance to protein adhesion and potent bacteria-eradicating ability that were maintained after water-aging for 180 days. The 3MPC + 1.5DMAHDM composite strongly deterred protein adhesion and diminished biofilm viability. It decreased the biofilm CFU by three orders of magnitude compared to a commercial composite, while possessing mechanical properties matching those of the commercial composite. The resistance to protein adhesion and the bacteria-eradicating ability were not reduced after water-aging for 180 days, demonstrating the durability and long-term benefits because of the covalent bonding of MPC and DMAHDM in the polymeric matrix. The MPC-DMAHDM composite with long-lasting resistance to protein adhesion and potent bacteria-eradicating capabilities represents a promising development for a wide range of dental restorations to inhibit caries and protect tooth structures.
